# In vitro antifungal susceptibility of Candida albicans isolates from oral cavities of patients infected with human immunodeficiency virus in Ethiopia

**DOI:** 10.1186/1742-4690-9-S1-P44

**Published:** 2012-05-25

**Authors:** Nasir Tajure Wabe, Jemal Hussein, Sultan Suleman, Kedir Abdella

**Affiliations:** 1 Jimma University, Jimma, Ethiopia

## Summary objective

Oral Candidiasis is the most common HIV related oral lesion. Most patients are infected with a strain originally present as a commensal of the oral cavity. The chronic use of antifungal agents, in the treatment of candidiasis mainly in HIV/AIDS patients leads to the selection of strain resistant to this therapy. The objective of this study was to evaluate the in vitro susceptibility of Candida albicans to commonly used antifungal agents in Ethiopia.

## Methods

In vitro susceptibility tests were performed using the broth microdilution method following the National Committee for Clinical Laboratory Standards (NCCLS) M27-A guidelines. Data were then analyzed using SPSS for windows version 16.0. Tests of proportions were done with Chi-Square, and a p value of <0.05 was considered as statistically significant.

## Results

A total of 42 oral C.albicans isolates from HIV-infected patients were included in this study. Forty one (97.7%) of all isolates were determined fully susceptible to amphotericin B, 40 (95.3%) to nystatin, and 39 (92.9%) to ketoconazole and miconazole. On the other hand, the isolates showed highest rates of resistance against fluconazole (11.9%) relatively. There was little difference in the antifungal susceptibilities of C.albicans isolated from patients who had a history of previous antifungal therapy compared with those who had not received antifungal treatment.

## Conclusion

The in vitro antifungal susceptibility testing of C.albicans in this study showed relatively high resistance to commonly used azoles. As with the prescribing of any antimicrobial agent, the use of a systemic antifungal drug must be justified. Efforts must be maintained to avoid inappropriate or unnecessary prescribing of these antifungal.

